# SOCIABILITY AMONG PERSONS LIVING WITH DEMENTIA IN A CREATIVE GROUP STORYTELLING CONTEXT

**DOI:** 10.1093/geroni/igz038.3204

**Published:** 2019-11-08

**Authors:** Kyong Hee Chee, Seoyoun Kim, Olga Gerhart, Sara Caldwell

**Affiliations:** 1 Texas State University, San Marcos, Texas, United States; 2 Texas State University, San Marcos, United States

## Abstract

Considering healthcare costs related to Alzheimer’s Disease and related dementias, shifting attention to the relatively malleable abilities of persons living with dementia holds promise for improving their well-being while reducing care burden. Defined as the ability to successfully interact with others, social intelligence is found to benefit well-being. Nevertheless, no known prior study has examined social intelligence among persons living with dementia. The purpose of this study is, therefore, to fill this gap by identifying the themes of social intelligence in this group. We used video-recorded data from an arts-based, creative group storytelling program (TimeSlips) that we implemented at Silverado Onion Creek Memory Care Community (currently, The Auberge) in Austin. The program is designed for persons living with dementia and involves a facilitator encouraging participants to use their imagination to collectively create a story from a staged picture. We offered 6 weekly sessions with 4 small groups of their residents (N = 26) in fall 2018 and spring 2019, and videotaped the sessions. Three researchers open-coded how participants interacted during storytelling sessions, and then met to reach consensus concerning verbal and non-verbal indicators of social intelligence. Major themes that emerged from our analysis are social awareness, initiating social interactions, and social diplomacy. Our findings suggest that those with lower cognitive function scores do not necessarily lack sociability. These findings add to social intelligence and dementia literatures, with potential implications for future research that can investigate the relationship between sociability and well-being among persons living with dementia.

